# Non-high-density lipoprotein cholesterol to high-density lipoprotein cholesterol ratio (NHHR) and hypertension in American adults: a NHANES cross-sectional study

**DOI:** 10.3389/fphys.2024.1398793

**Published:** 2024-08-13

**Authors:** Jiabei Wu, Jinli Guo

**Affiliations:** ^1^ Shanxi Medical University, Taiyuan, China; ^2^ The Second Hospital of Shanxi Medical University, Taiyuan, China

**Keywords:** lipid ratio, Non-HDL-C/HDL-C (NHHR), NHANES, hypertension, cross-sectional study

## Abstract

**Objectives:**

The relationship between non-high-density lipoprotein cholesterol to high-density lipoprotein cholesterol ratio (NHHR) and hypertension remains uncertain, warranting further investigation. This study aims to elucidate the association between NHHR and hypertension.

**Methods:**

A comprehensive cross-sectional stratified survey involving 30,602 participants aged 20 years and older was conducted using the National Health and Nutrition Examination Survey (NHANES) dataset from 2001 to 2018. NHHR was calculated as [total cholesterol (TC) - high-density lipoprotein cholesterol (HDL-C)]/HDL-C. The relationship between NHHR and hypertension was examined using weighted multiple linear regression, smooth curve fitting, hierarchical analysis, and interaction testing.

**Results:**

The mean age of participants was 49.82 ± 17.64 years, with 15,266 women included. The average NHHR was 2.94 ± 0.56. A positive correlation between NHHR and hypertension was observed. Stratification of NHHR into quartiles, in the fully adjusted Model 3, revealed that individuals in the highest NHHR quartile had a 60% increased risk of hypertension for each unit increase in NHHR compared to those in the lowest quartile. Interaction tests indicated that the relationship between NHHR and hypertension remained consistent across subgroups, except for gender, age, education, and smoking status, which influenced this association.

**Conclusion:**

Analysis of NHANES data from 2001 to 2018 demonstrated a consistent positive association between NHHR and hypertension. NHHR may provide potential assistance in hypertension prevention and diagnosis.

## 1 Introduction

A robust direct correlation between blood pressure levels and the risk of clinical complications and mortality was first observed in studies involving adults as early as the 1920s (Build and Blood Pressure Study, 1959. Volume 1, 1960), and later confirmed in research on heart disease in the 1960s (Levy, 1981). The cumulative long-term impacts of hypertension are linked to a broad spectrum of detrimental health outcomes, with nearly half of all adverse cardiac, cerebral, and renal events attributed to hypertension (Sliwa et al., 2011; Rapsomaniki et al., 2014). These outcomes include left ventricular hypertrophy (Escobar, 2002), cerebral hemorrhage, renal failure (Doyle, 1992), retinopathy, stroke (Chatterjee et al., 2002), and eclampsia during pregnancy (Au et al., 2017; Antza et al., 2018). The annual rise in hypertension incidence has accelerated over the past decade, with an estimated 1.13 billion individuals worldwide living with hypertension (Williams et al., 2018). As the global population ages and obesity rates climb, the prevalence of hypertension is also on the rise, with projections indicating that by 2025, one-third of the global population will be affected by hypertension (Oliveros et al., 2020). Despite increased awareness of hypertension in recent years, poor adherence to chronic disease treatment remains a global challenge (Vrijens et al., 2017). Hypertension manifests differently across genders, regions, and economic circumstances (Mills et al., 2020). In low- and middle-income nations, the prevalence of uncontrolled hypertension is high and increasing (Sun et al., 2022). Furthermore, hypertension is strongly associated with premature mortality (Roth et al., 2015). In 2015, 874 million adults had a systolic blood pressure (SBP) of 140 mmHg or higher, and there has been a significant increase in the number of related deaths (Forouzanfar et al., 2017), imposing a significant global burden.

Non-High-Density Lipoprotein Cholesterol (Non-HDL-C) is considered a pivotal contributor to cardiovascular (CV) disease (Carr et al., 2019). High-Density Lipoprotein Cholesterol (HDL-C) exhibits an inverse relationship with CV events, with a 2%–3% reduction for every 1 mg/dL increase, exerting CV protective effects primarily through cholesterol transfer reversal, anti-inflammatory, antioxidant, anti-apoptotic, and vasodilatory mechanisms (Badimón et al., 2010; Grundy et al., 2019; Sun et al., 2019). The non-high-density lipoprotein cholesterol to high-density lipoprotein cholesterol ratio (NHHR) is a novel lipid ratio utilized to evaluate CV disease risk (Qi et al., 2024), introduced by Chinese scholars in 2022 following a longitudinal study involving 15,000 individuals. Currently, a series of epidemiological investigations have been conducted on non-traditional lipid indicators such as NHHR and Non-HDL-C. When it comes to evaluating and forecasting diabetes, non-traditional lipid measurements often perform better than standard lipid indicators (Sheng et al., 2022). The ability of conventional lipid parameters to evaluate and detect risks associated with a variety of diseases has been improved by the use of non-traditional lipid parameters as new markers for endocrine, CV, digestive, urinary, and respiratory system problems.

Two important risk factors for atherosclerotic CV disease are hypertension and dyslipidemia (Xie et al., 2022). Obesity, lipid-related indicators, and hypertension have been found to be significantly correlated in recent research (Gui et al., 2024). According to some studies, the risk of developing hypertension increases when total cholesterol (TC), triglycerides (TG), Low-Density Lipoprotein Cholesterol (LDL-C), or Non-HDL-C are raised and HDL-C is lowered (Hunt et al., 1991; Otsuka et al., 2016; Sun et al., 2017; He et al., 2021).

However, the majority of research has concentrated on the correlation between a single lipid indicator and hypertension, leaving an incomplete understanding of the link between the combined effects of various lipid indicators and hypertension. Clarifying the association between NHHR and hypertension holds significant clinical importance in advancing patients’ physical and mental well-being, reducing societal burdens, and judiciously allocating medical resources. Hence, this study analyzed data from the National Health and Nutrition Examination Survey (NHANES) spanning 2001 to 2018 to investigate the relationship between NHHR and hypertension.

## 2 Materials and methods

### 2.1 Study participants

**FIGURE 1:**
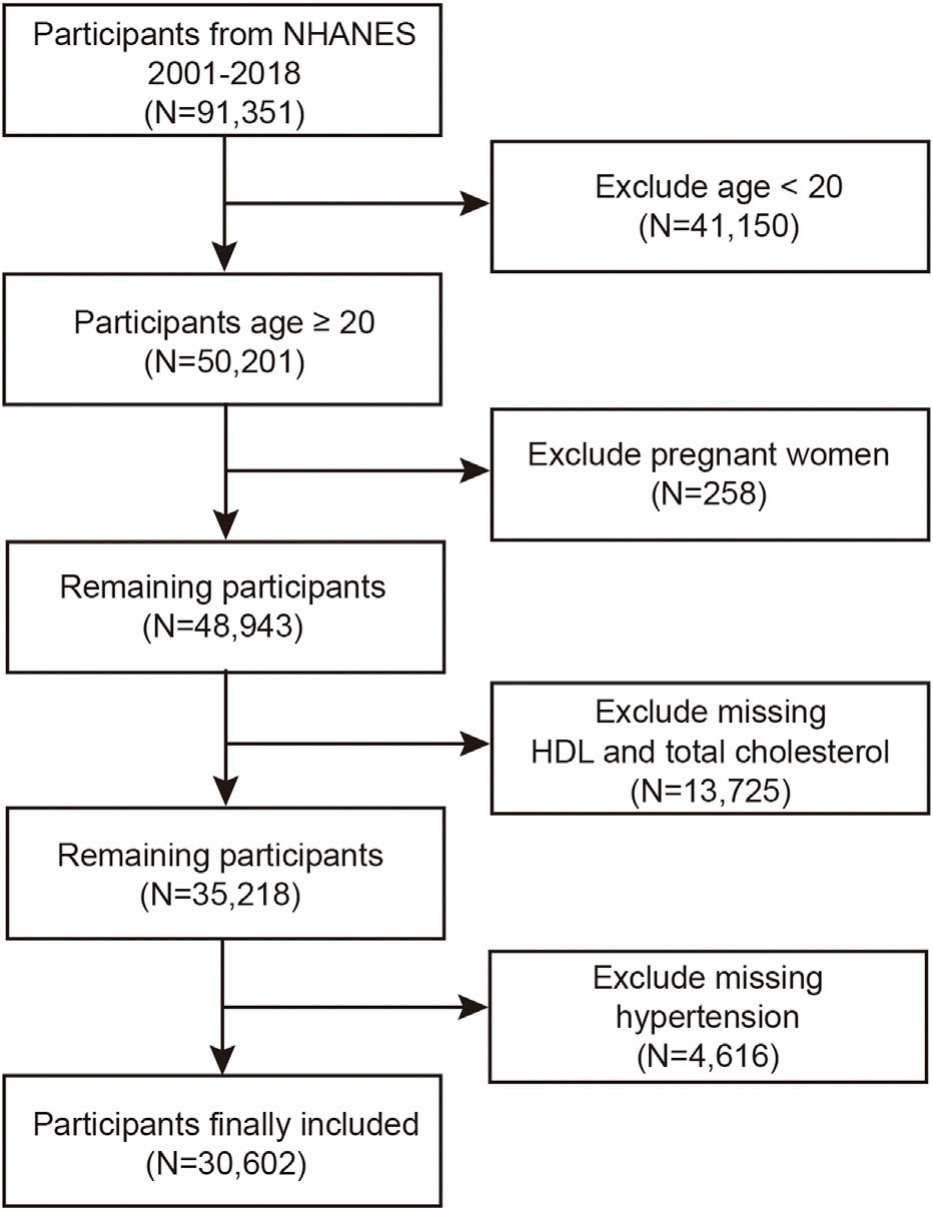
Flow chart of participants selection.

Using information from the biannual NHANES, we carried out a thorough cross-sectional, stratified study. This dataset comprises a diverse range of information, encompassing potential risk factors and nutrient levels, thereby facilitating a thorough exploration of the factors influencing the health of the U.S. population. All participants in the NHANES study adhered strictly to protocols sanctioned by the Ethics Review Board of the National Center for Health Statistics (NCHS ERB) and completed informed consent forms as required (Guo et al., 2022). For this research, we performed an analysis using data from nine 2-year NHANES cycles spanning from 2001 to 2018. Out of the initial 91,351 participants, we excluded 41,150 individuals under 20 years of age, 258 participants who were pregnant, and 13,725 individuals lacking HDL-C and TC data. Furthermore, 4,616 individuals were excluded due to missing data concerning hypertension. Ultimately, the study involved 30,602 participants (Figure 1).

### 2.2 Exposure variable

The NHHR represents a novel lipid ratio, specifically defined as the ratio of Non-HDL-C to HDL-C (Wang et al., 2022; Qing et al., 2024). Non-HDL-C is derived by subtracting HDL-C from TC (Wu et al., 2019). HDL-C is quantified in a distinct manner to differentiate it from other cholesterol constituents. This process entails the conversion of cholesterol esters into HDL-C, generating a discernible pigment for quantification. The laboratory technique employed for total cholesterol measurement is based on an enzymatic assay.

### 2.3 Outcome variable

In this study, hypertension serves as the outcome variable, as per the 2017 American College of Cardiology/American Heart Association Guideline for the Prevention, Detection, Evaluation, and Management of High Blood Pressure in Adults, and is characterized by (Whelton et al., 2018): ① systolic blood pressure (SBP) ≥140 mmHg and/or diastolic blood pressure (DBP) ≥90 mmHg; ② the use of prescribed medications for hypertension; ③ self-reported hypertension. Meeting any one of these criteria signifies the presence of hypertension (Wu et al., 2023). Blood pressure assessments were conducted during the NHANES examination. Following a period of seated rest for 5 min, blood pressure was measured thrice by a trained examiner using a mercury sphygmomanometer. If necessary, a fourth reading was taken. Subsequently, the SBP and DBP values were averaged (Shen et al., 2021, pp. 2007–2014).

### 2.4 Covariates

For this investigation, potential confounding variables that might influence the association between NHHR and hypertension were collected. The selected variables for analysis included: age (years), gender, ethnicity (categorized as Mexican American, other Hispanic, non-Hispanic White, non-Hispanic Black, or other race), level of education (classified as less than high school, high school, or more than high school), marital status, smoking status (determined by inquiring whether participants had ever smoked at least 100 cigarettes in their lifetime) (yes/no), presence of hypertension, average daily alcohol consumption (drinks/day), family income-to-poverty ratio (Family PIR), levels of HDL-C (mg/dL) and TC (mg/dL), BMI (kg/m^2^), high cholesterol status, use of prescribed hypertension medications, NHHR, DBP (mmHg), SBP (mmHg), and diabetes status.

### 2.5 Statistical analysis

This study used frequencies (%) to represent categorical variables and mean and standard deviation to define continuous variables. A linear regression analysis was conducted to investigate the association between NHHR and hypertension. Model 1 represented an unadjusted model devoid of covariates, whereas Model two incorporated age, gender, and race as covariates. The fully adjusted Model three additionally adjusted for educational level, Family PIR, marital status, smoking habits, and alcohol intake. Using the software packages R version 4.3.3 (http://www.R-project.org) and EmpowerStats software (http://www.empowerstats.com), we examined the connection between NHHR and hypertension using weighted multiple logistic regression and smooth curve fitting methods. Tests of interaction and hierarchical analysis were then carried out.

## 3 Results

### 3.1 Characteristics of the study population

A total of 30,602 individuals were recruited for this study. NHHR was stratified into quartiles defined by the values 1.932, 2.662, and 3.648. Table 1 displays the baseline characteristics of the study population according to NHHR quartiles. The mean age of the participants was 49.82 ± 17.64 years, with 15,266 females (49.89%) included. The average NHHR was 2.94 ± 0.56. NHHR levels exhibited correlations with various factors including age, gender, race, education level, marital status, smoking habits, hypertension, high cholesterol, hypertension medication use, Family PIR, HDL-C, TC, alcohol consumption, BMI, DBP, SBP, hypertension status, and diabetes status, all of which were statistically significant (*p* < 0.05). Specifically, married non-Hispanic White males who are regular smokers, possess higher educational attainment, take prescription hypertension medications, have a BMI exceeding 30 kg/m^2^, and consume elevated levels of alcohol are more inclined to demonstrate elevated NHHR levels.

**TABLE 1:** Basic characteristics of participants by non-high-density lipoprotein cholesterol to high-density lipoprotein cholesterol ratio quartile.

Characteristics	NHHR				*p*-value.
	Q1 (≤1.932) N = 7,650	Q2 (1,932, 2.662) N = 7,622	Q3 (2.662, 3.648) N = 7,677	Q4 (≥3.648) N = 7,653	
Age(years)	49.58 ± 19.41	50.45 ± 18.37	50.21 ± 16.97	49.03 ± 15.53	<0.001
Gender, n (%)					<0.001
Male	2,777 (36.30)	3,355 (44.02)	4,184 (54.50)	5,020 (65.60)	
Female	4,873 (63.70)	4,267 (55.98)	3,493 (45.50)	2,633 (34.40)	
Race/ethnicity, n (%)					<0.001
Mexican American	836 (10.93)	1,062 (13.93)	1,368 (17.82)	1,549 (20.24)	
Other Hispanic	569 (7.44)	721 (9.46)	824 (10.73)	914 (11.94)	
Non-Hispanic White	3,288 (42.98)	3,253 (42.68)	3,198 (41.66)	3,333 (43.55)	
Non-Hispanic Black	2,051(26.81)	1,707(22.40)	1,455 (18.95)	1,032 (13.48)	
Other Race	906 (11.84)	879 (11.53)	832 (10.84)	825 (10.78)	
Education level, n (%)					<0.001
Less than high school	1,558 (20.39)	1,743 (22.88)	2,001 (26.08)	2,284 (29.88)	
High school	1,609 (21.06)	1,706 (22.40)	1,844 (24.03)	1,814 (23.73)	
More than high school	4,474 (58.55)	4,168 (54.72)	3,829 (49.90)	3,546 (46.39)	
Marital status, n (%)					<0.001
Married/Living with Partner	4,080 (53.35)	4,458 (58.51)	4,797 (62.55)	5,039 (65.85)	
Widowed/Divorced/Separated	1,829 (23.91)	1,709 (22.43)	1,647 (21.48)	1,551 (20.27)	
Never married	1,739 (22.74)	1,452 (19.06)	1,225 (15.97)	1,062 (13.88)	
Smoked at least 100 cigarettes, n (%)					<0.001
Yes	3,175 (41.50)	3,254 (42.69)	3,460 (45.07)	3,902 (50.99)	
No	4,475 (58.50)	4,368 (57.31)	4,217 (54.93)	3,751 (49.01)	
High blood pressure, n (%)					<0.001
Yes	2,537 (33.16)	2,722 (35.71)	2,840 (36.99)	2,804 (36.64)	
No	5,106 (66.75)	4,889 (64.14)	4,826 (62.86)	4,832 (63.14)	
High cholesterol, n (%)					<0.001
Yes	1,989 (29.34)	2,356 (35.02)	2,686 (39.53)	3,214 (48.67)	
No	4,751 (70.07)	4,323 (64.25)	4,063 (59.80)	3,337 (50.54)	
Taking prescription for hypertensions, n (%)					<0.001
Yes	2,272 (89.55)	2,428 (89.20)	2,482 (87.39)	2,367 (84.42)	
No	261 (10.29)	290 (10.65)	356 (12.54)	436 (15.55)	
Average alcohol consumption (drinks/day)	3.94 ± 36.02	3.39 ± 28.97	4.91 ± 44.87	4.04 ± 27.55	<0.001
Family PIR	2.65 ± 1.65	2.59 ± 1.63	2.50 ± 1.61	2.38 ± 1.59	<0.001
HDL-C (mg/dL)	69.40 ± 16.53	55.65 ± 10.91	47.61 ± 8.72	38.71 ± 7.82	<0.001
DBP (mmHg)	67.94 ± 12.37	68.87 ± 12.25	70.65 ± 12.49	72.89 ± 12.18	<0.001
SDP (mmHg)	122.48 ± 19.40	123.15 ± 18.11	124.45 ± 17.64	125.98 ± 17.24	<0.001
TC (mg/dL)	169.98 ± 34.56	182.64 ± 34.66	195.31 ± 34.84	221.74 ± 42.74	<0.001
BMI (kg/m2)	26.43 ± 6.43	28.74 ± 6.82	30.17 ± 6.70	31.03 ± 6.22	<0.001
Hypertension, n (%)	85.92 ± 11.24	98.00 ± 12.40	105.04 ± 13.73	114.94 ± 16.12	<0.001
Yes	2,974 (38.88)	3,187 (41.81)	3,337 (43.47)	3,403 (44.47)	<0.001
No	4,676 (61.12)	4,435 (58.19)	4,340 (56.53)	4,250 (55.53)	<0.001
NHHR	1.49 ± 0.32	2.29 ± 0.21	3.12 ± 0.28	4.87 ± 1.41	<0.001
Diabetes, n (%)					<0.001
Yes	940 (12.29)	943 (12.37)	1,020 (13.29)	1,050 (13.72)	
No	6,710 (87.71)	6,679 (87.63)	6,657 (86.71)	6,603 (86.28)	

Mean ± SD, for continuous variables: the *p*-value was analyzed via ANOVA. (%) for categorical variables: the *p*-value was analyzed via the weighted chi-square test.

Abbreviation: Family PIR, the ratio of family income to poverty; BMI, body mass index; HDL-C, High-Density Lipoprotein Cholesterol; TC, total cholesterol; NHHR, non-high-density lipoprotein cholesterol to high-density lipoprotein cholesterol ratio; SBP, systolic blood pressure; DBP, diastolic blood pressure.

### 3.2 Association between NHHR and hypertension

**FIGURE 2:**
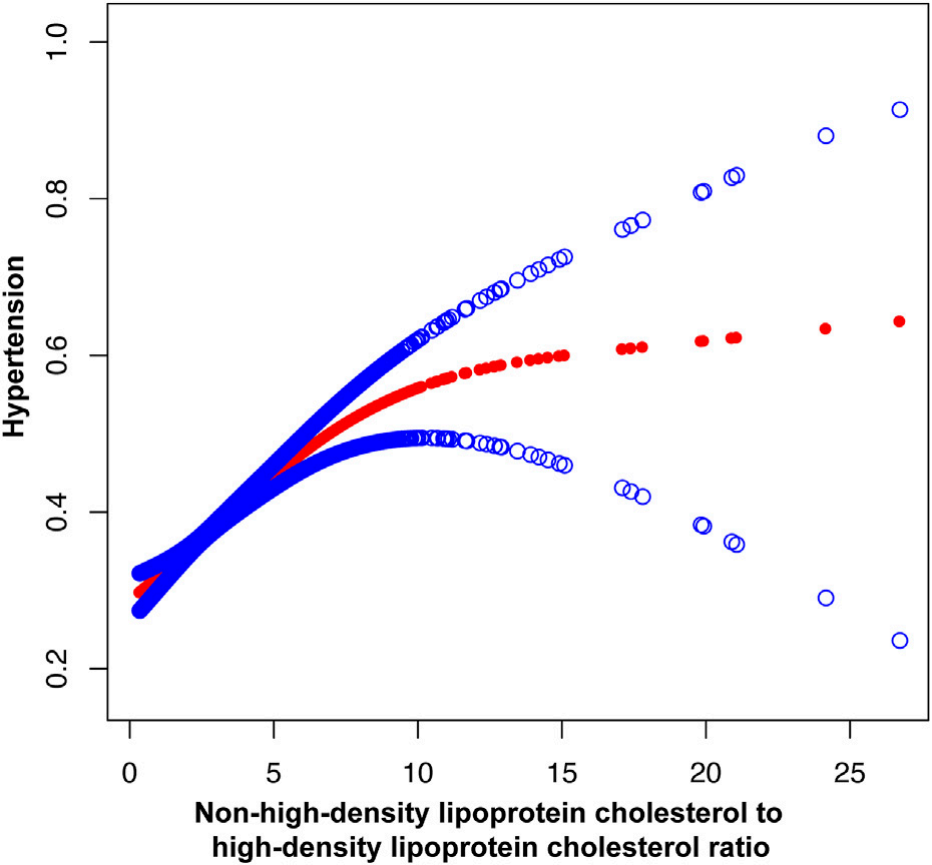
The association between non-high-density lipoprotein cholesterol to high-density lipoprotein cholesterol ratio and hypertension. Age, gender, race, educational level, Family PIR, marital status, smoking status and drinking alcohol status were adjusted.

**FIGURE 3:**
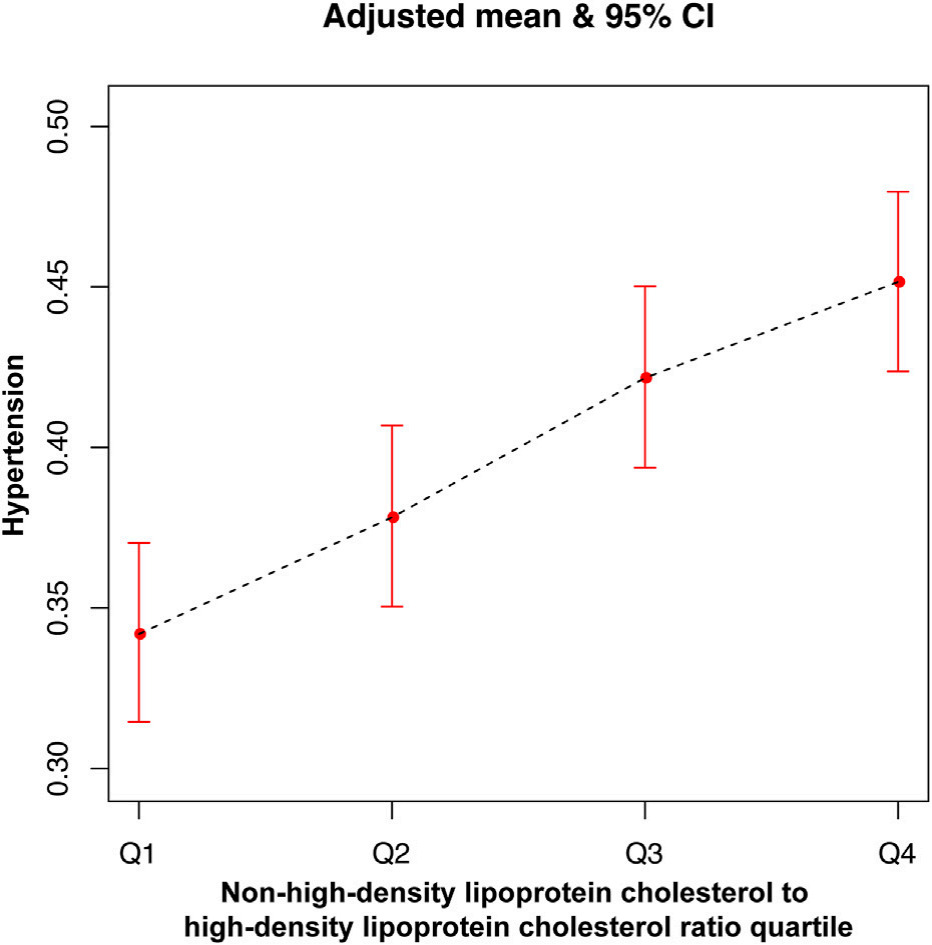
The association between non-high-density lipoprotein cholesterol to high-density lipoprotein cholesterol ratio quartile and hypertension.

The multifactor logistic regression models analyzing the relationship between NHHR and hypertension are presented in Table 2. NHHR, treated as a continuous variable, exhibited a positive correlation with hypertension across all three models. In Model 3, with each incremental unit rise in NHHR, individuals experienced a 13% heightened likelihood of developing hypertension (OR = 1.13, 95% CI: 1.11, 1.16). The respective OR values for Model 1 and Model 2 are 1.06 (1.04, 1.07) and 1.15 (1.13, 1.17). The smooth curve fitting further underscored the significant positive association between NHHR and hypertension. In Figure 2, the horizontal coordinate represents the range of NHHR, the vertical coordinate is the risk of developing hypertension, the red dotted line indicates the result of performing a smooth curve fit between NHHR and hypertension, and the blue dotted line indicates the 95% confidence interval (95% CI) of the fit. Figure 3 shows the results of smoothed curve fitting between NHHR and hypertension under the fully adjusted model after converting NHHR from a continuous variable to a categorical variable. Upon stratifying NHHR into quartiles, the fully adjusted model revealed that individuals within the highest NHHR quartile faced a 60% increased risk of hypertension for each unit increment in NHHR compared to those in the lowest quartile (OR = 1.60, 95% CI: 1.44–1.77).

**TABLE 2:** Association of non-high-density lipoprotein cholesterol to high-density lipoprotein cholesterol ratio with hypertension.

	OR (95% CI)		
Exposure	Model 1	Model 2	Model 3
	(n = 30,602)	(n = 30,602)	(n = 17,899)
NHHR	1.06 (1.04, 1.07)	1.15 (1.13, 1.17)	1.13 (1.11, 1.16)
NHHR quartile			
Quartile 1	Reference	Reference	Reference
Quartile 2	1.13 (1.06, 1.21)	1.17 (1.09, 1.27)	1.16 (1.05, 1.28)
Quartile 3	1.21 (1.13, 1.29)	1.38 (1.27, 1.49)	1.38 (1.25, 1.53)
Quartile 4	1.26 (1.18, 1.34)	1.70 (1.57, 1.83)	1.60 (1.44, 1.77)
P for trend	<0.001	<0.001	<0.001

Model 1= No covariates were adjusted.

Model 2 = Age, gender, and race were adjusted.

Model 3 = Age, gender, race, educational level, Family PIR, marital status, smoking status and drinking alcohol status were adjusted.

Abbreviation: OR, odds ratio; 95% CI, 95% confidence interval; Family PIR, the ratio of family income to poverty; NHHR, non-high-density lipoprotein cholesterol to high-density lipoprotein cholesterol ratio.

In sensitivity analysis, NHHR, was converted from a continuous variable to a categorical variable (quartile).

### 3.3 Subgroup analysis

**FIGURE 4:**
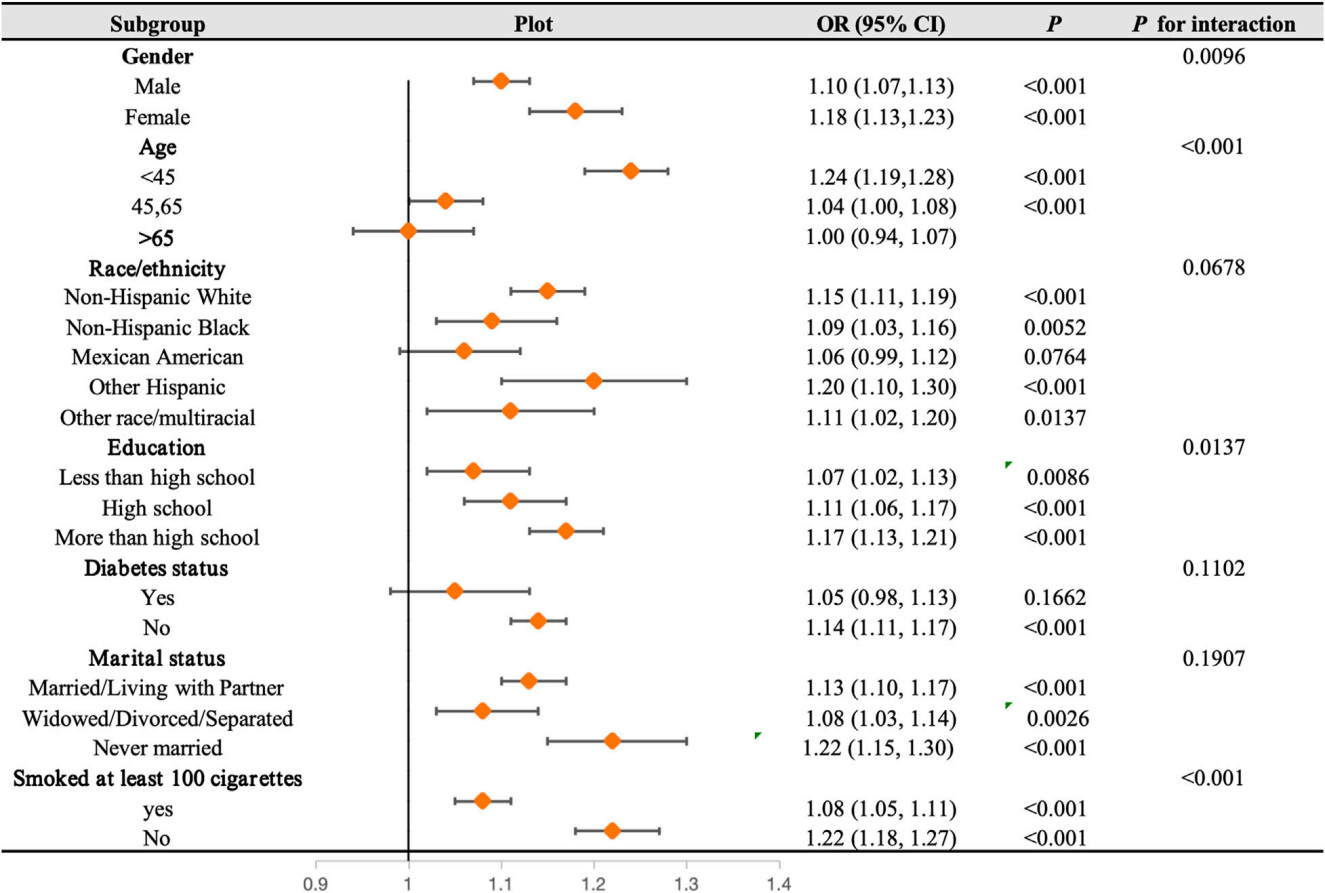
Subgroups analyses. In the subgroup analysis stratified by age, gender, race, educational level, Family PIR, marital status, smoking status, and drinking alcohol status, the model is not adjusted for age, gender, race, educational level, Family PIR, marital status, smoking status, and drinking alcohol status.

In order to delve deeper into the potential association between NHHR and hypertension across diverse conditions, a subgroup analysis was conducted in this study (Figure 4). Findings from the interaction test indicated that the correlation between NHHR and hypertension remained consistent across race, diabetes status, and marital status, displaying no significant impact (*p* for interaction > 0.05). However, this relationship exhibited variability concerning gender, age, education, and smoking status. Specifically, the link between NHHR and hypertension was notably influenced by gender, age, education, and smoking habits, with younger individuals, those with higher educational levels, women, and smokers more likely to affect this association.

## 4 Discussion

In order to look into possible connections between hypertension and NHHR, we evaluated NHANES data from 2001 to 2018. The results of the smoothed curve fitting further showed a strong positive correlation between hypertension and NHHR. In the fully adjusted model, each one-unit increase in the highest NHHR quartile (≥3.648) was associated with a 60% higher likelihood of developing hypertension compared to Q1 (≤1.932). Subgroup analysis indicated that the relationship between NHHR and hypertension remained consistent across subgroups, except for gender, age, education, and smoking status (*p* for interaction > 0.05). This suggests that gender, age, education, and smoking status influence the association between NHHR and hypertension. Monitoring NHHR can offer improved early detection of high blood pressure and aid in identifying individuals at risk of hypertension. This proactive approach can facilitate preventive measures, such as dietary and lifestyle modifications, to mitigate the risk of developing hypertension (Ding et al., 2020). When necessary, healthcare professionals can devise personalized treatment plans for patients and provide effective medical guidance.

Available studies have shown that NHHR is a lipid indicator with the ability to predict atherosclerosis (Wang et al., 2022; 2024), which triggers vascular remodeling and further contributes to an increase in blood pressure (Hurtubise et al., 2016). Our study suggests that NHHR may be a contributing factor in the development of hypertension. An early study by McLaughlin et al., in 2003 focused on the relationship between the TG/HDL-C ratio and cardiometabolic risk in 258 overweight volunteers without prior hypertension diagnoses. The findings suggested that the TG/HDL-C ratio may elevate their CV risk (McLaughlin et al., 2003). A cohort study of men found that elevated levels of Non-HDL-C were associated with an increased risk of hypertension in Japanese men (Otsuka et al., 2016). A 2018 study in China on predicting hypertension risk highlighted that lipid accumulation measurements provide a comprehensive understanding of hypertension risk related to changes in body fat distribution, offering valuable insights into hypertension risk (Wang et al., 2018). Dyslipidaemia may help identify women at risk of hypertension, suggests a study among Middle Eastern women (Tohidi et al., 2012). A prospective cohort study in the UK Biobank indicated that the increased risk of death associated with very high HDL-C was present in hypertensive patients (Chen et al., 2023).

The mechanistic link by which NHHR can serve as a lipid marker for predicting hypertension is unclear, but previous studies have provided several hypotheses. First, dyslipidemia may damage the endothelium of blood vessels, thereby interfering with blood pressure regulation (Creager et al., 1990). Second, plasma lipids enhance α1-adrenergic receptor pressor sensitivity, impair baroreflex function, and are linked to elevated blood pressure (Gadegbeku et al., 2006). Finally, dyslipidemia leads to increased blood pressure by inducing insulin resistance, which in turn activates the renin-angiotensin system (McGill et al., 2009). Investigating the impact of excess lipid supply on cardiac function in mice displaying signs of hyperlipidemia and hypertension after a high-fat diet, Qingxun Hu’s team discovered that excess lipid supply fosters an intracellular environment conducive to Drp1 acetylation, with Drp1 potentially playing a pivotal role in cardiac dysfunction induced by lipid overload (Hu et al., 2020). Research on novel clinical hypertension biomarkers has been conducted extensively, and this is certainly a promising line of research (Zhang and Sun, 2022).

Our study showed in subgroup analyses that gender affects the association between lipids and hypertension, especially for women. Kaneva et al. studied gender differences in obesity and hypertension and showed that among overweight and obese people, women with hypertension exhibited higher lipid levels compared to normotensive women, while there was no significant difference in men (Kaneva and Bojko, 2023). Previous studies have examined the influence of education on overall CV risk in hypertensive outpatients, revealing that lower educational attainment and limited literacy are independent predictors of inadequate knowledge and control of hypertension (Pandit et al., 2009; Di Chiara et al., 2017). Similarly, in our study, educational attainment exerted varying degrees of influence on the relationship between NHHR and hypertension. Another contributing factor is smoking, which raises the risk of vascular damage by producing more free radicals, endothelial damage, sympathetic tone, platelet stickiness and reactivity, and an increase in arterial pressure. Smokers are also more likely to experience severe hypertension (Sleight, 1993; Virdis et al., 2010). These findings corroborate our findings.

Our research boasts several notable strengths. We conducted a large representative sample study, utilized data sets from 2001 to 2018, adjusted for confounding covariates to enhance result robustness, and conducted sensitivity analyses. This is the first study that we are aware of that looks at the connection between NHHR and hypertension in a US population. There are certain restrictions, though. First of all, it is difficult to determine the causal links between exposure factors and outcomes because of the cross-sectional design. Therefore, the findings of this study should be interpreted cautiously, and further clarification of causality necessitates multiple prospective studies. Secondly, despite including several covariates, not all potential confounding variables related to NHHR, and hypertension were accounted for in the study. While we adjusted for potentially confounding factors, the presence of residual confounding factors cannot be entirely ruled out. Further research is warranted to identify additional novel lipid-related biomarkers and assess the reliability of these biomarkers, or even different combinations thereof, in clinically predicting hypertension risk.

## 5 Conclusion

We examined the association between NHHR and hypertension utilizing NHANES data spanning from 2001 to 2018. Our findings reveal a positive correlation between the two variables. Subgroup analysis indicates that the relationship between NHHR and hypertension remained consistent across all subgroups, except for gender, age, education, and smoking status. This discovery may offer novel lipid-related biomarkers for proactive hypertension screening, thereby playing a significant role in mitigating the onset of hypertension.

## Data Availability

The original contributions presented in the study are included in the article/supplementary material, further inquiries can be directed to the corresponding author.

## Abbreviations

(NHHR), Non-high-density lipoprotein cholesterol to high-density lipoprotein cholesterol ratio; (NHANES), National Health and Nutrition Examination Survey; (BMI), Body mass index; (Family PIR), family income-to-poverty ratio; (HDL-C), High-Density Lipoprotein Cholesterol; (LDL-C), Low-Density Lipoprotein Cholesterol; (Non-HDL-C), Non-High-Density Lipoprotein Cholesterol; (CV), Cardiovascular; (TC) Total cholesterol; (TG), Triglycerides; (SBP), Systolic blood pressure; (DBP), Diastolic blood pressure; (95% CI), 95% confidence interval.
